# The Role of Nucleus Accumbens Core/Shell in Sleep-Wake Regulation and their Involvement in Modafinil-Induced Arousal

**DOI:** 10.1371/journal.pone.0045471

**Published:** 2012-09-19

**Authors:** Mei-Hong Qiu, Wei Liu, Wei-Min Qu, Yoshihiro Urade, Jun Lu, Zhi-Li Huang

**Affiliations:** 1 State Key Laboratory of Medical Neurobiology, Shanghai Medical College of Fudan University, Shanghai, China; 2 Department of Pharmacology, Shanghai Medical College of Fudan University, Shanghai, China; 3 Institutes of Brain Science, Fudan University, Shanghai, China; 4 Department of Molecular Behavioral Biology, Osaka Bioscience Institute, Suita, Osaka, Japan; 5 Department of Neurology, Beth Israel Deaconess Medical Center and Harvard Medical School, Boston, Massachusetts, United States of America; Imperial College London, United Kingdom

## Abstract

**Background:**

We have previously shown that modafinil promotes wakefulness via dopamine receptor D_1_ and D_2_ receptors; however, the locus where dopamine acts has not been identified. We proposed that the nucleus accumbens (NAc) that receives the ventral tegmental area dopamine inputs play an important role not only in reward and addiction but also in sleep-wake cycle and in mediating modafinil-induced arousal.

**Methodology/Principal Findings:**

In the present study, we further explored the role of NAc in sleep-wake cycle and sleep homeostasis by ablating the NAc core and shell, respectively, and examined arousal response following modafinil administration. We found that discrete NAc core and shell lesions produced 26.5% and 17.4% increase in total wakefulness per day, respectively, with sleep fragmentation and a reduced sleep rebound after a 6-hr sleep deprivation compared to control. Finally, NAc core but not shell lesions eliminated arousal effects of modafinil.

**Conclusions/Significance:**

These results indicate that the NAc regulates sleep-wake behavior and mediates arousal effects of the midbrain dopamine system and stimulant modafinil.

## Introduction

The nucleus accumbens (NAc) located in the ventral striatum is a part of the basal ganglia and limbic system. The NAc plays an important role in reward and addiction as well as aggression and fear [1]–[3]. Based on the neural make-up, projections and functions of the NAc [4]–[10], the NAc is divided into the core and shell.

Our previous lesion studies showed that NAc lesions by ibotenic acid caused a significant increase in the amount of wakefulness by an average of 27% across day-night. The wake increase was accompanied by sleep fragmentation (frequent sleep-wake transition and short sleep bout duration) [11]. These results reveal a novel role of the NAc in sleep-wake regulation. However, because the NAc lesions were mostly confined in the NAc core and in light of a recent study showing that NAc shell adenosine A_2A_ receptors mediated arousal effects of caffeine [12], it is crucial to investigate if the NAc shell is also involved in sleep-wake regulation.

Modafinil is one of most popular stimulants [13], [14]. Dopamine transporter (DAT) knockout mice show elevated extracellullar dopamine and a blunt arousal response following modafinil but not caffeine administration [15], indicating that dopamine system mediates arousal effects of modafinil. Our recent study further demonstrated that both dopamine D_1_ and D_2_ receptors were involved in regulation of modafinil-induced arousal [16]. However, the neuronal circuitry that mediates arousal of dopamine and modafinil has not been identified. We hypothesized that the NAc innervated by the ventral tegmental area (VTA) dopaminergic neurons mediates arousal induced by modafinil.

**Figure 1:**
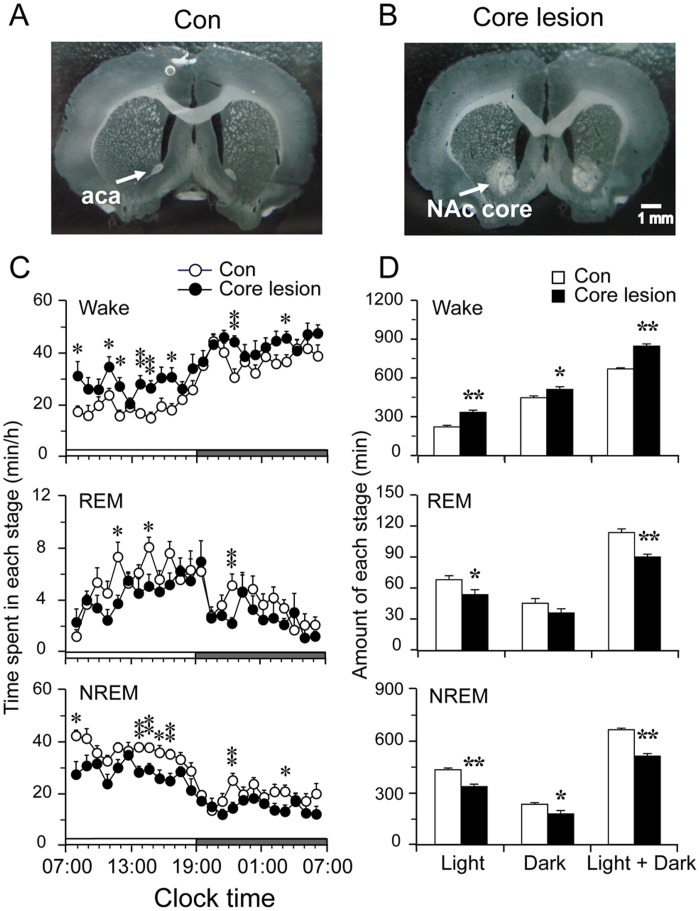
NAc core lesion increases wakefulness. A and B: photographs of representative coronal sections from a control (A) and a lesion case (B), the pale parts in B show the lesion in NAc core. Scale bar: 1 mm. aca: anterior commissure, anterior part. C: The hourly amount of wakefulness, REM and NREM sleep of control and NAc core lesioned group. Each circle represents the hourly mean ± SEM of each stage. D: Total time spent in wakefulness, REM and NREM sleep during the light and dark periods and over the 24-h day. *p<0.05, **p<0.01.

In the present study, we selectively lesioned NAc core and shell in rats, and examined their basal sleep-wake changes and sleep rebound after 6 hrs sleep deprivation (SD) and arousal response following modafinil administration. We found that both NAc core and shell lesions increased wakefulness but core lesions had a bigger arousal effect, and that both lesions reduced sleep rebound after 6-hr SD. NAc core lesions but not NAc shell lesions blocked arousal response to modafinil.

## Materials and Methods

### Animals

Pathogen-free adult male Sprague Dawley rats (275–300 g) were obtained from the Laboratory Animal Center, Chinese Academy of Sciences (Shanghai, China). The animals were housed in individual cages at a constant temperature (22±0.5°C) with a relative humidity (60±2%) on an automatically controlled 12∶12 light/dark cycle (light on at 7 A.M.), and had free access to food and water. The experimental protocols were approved by the Committee on the Ethics of Animal Experiments of the University of Fudan, Shanghai medical college (Permit Number: 20110307-049) and the Animal Research Committee of Osaka Bioscience Institute. Every effort was made to minimize the number of animals used and any pain and discomfort experienced by the subjects.

**Figure 2:**
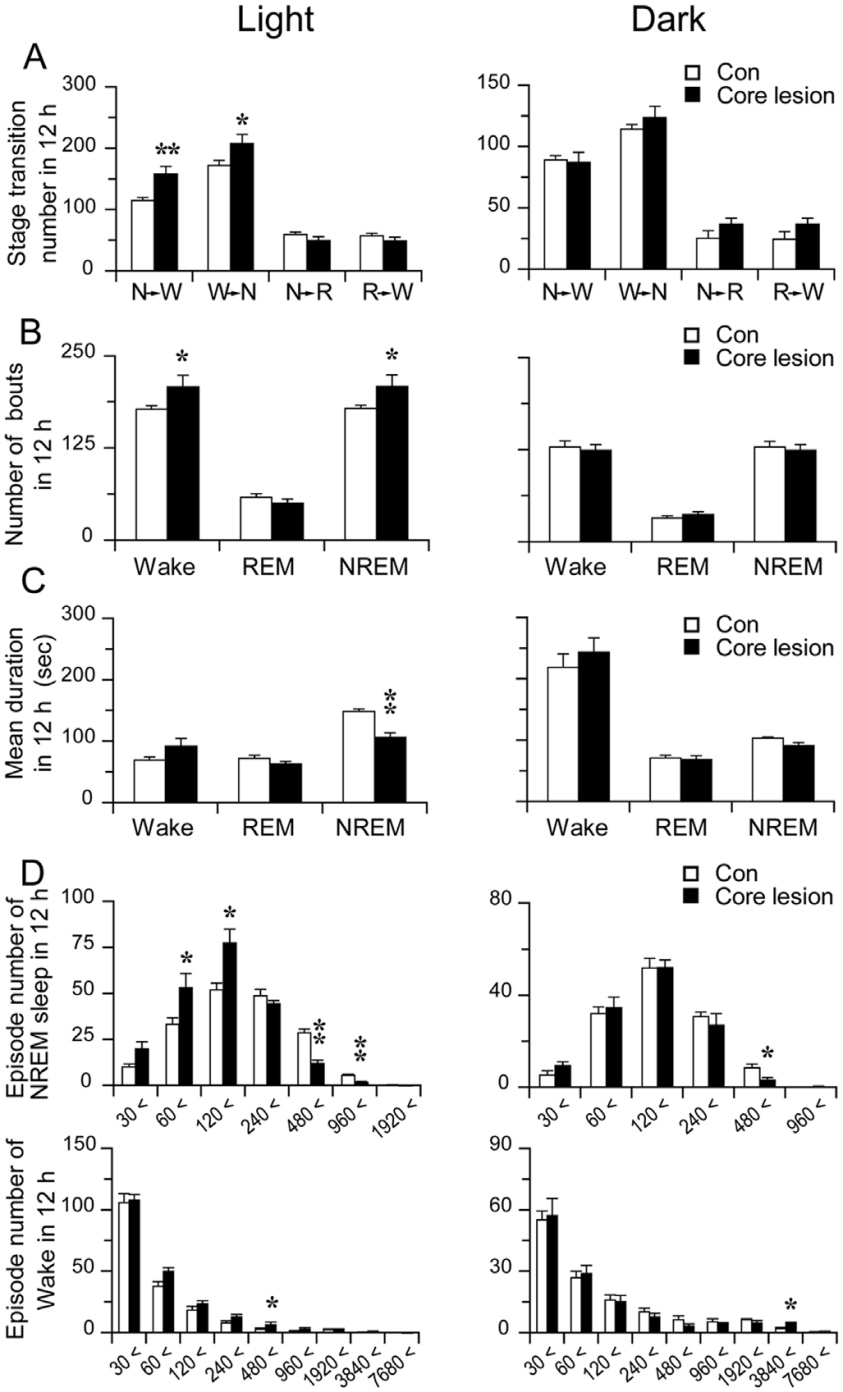
NAc core lesion causes sleep fragmentation. A: Sleep-wake stage transitions during the light and dark period (N, W and R represent NREM sleep, wakefulness and REM sleep, respectively). B and C: The number of bouts (B) and mean durations (C) during the light and dark periods. D: Distribution of number of NREM sleep and wake bouts across different episode durations during light and dark period. *p<0.05, **p<0.01.

### Neurotoxin Injection

Under chloral hydrate anesthesia (10% in saline, 360 mg/kg), a burr hole was made and a fine glass pipette (1 mm glass stock, tapering slowly to a 10–20 micron tip) containing 1% ibotenic acid (Sigma, St Louis, MO, USA) was lowered to the NAc core (AP = +1.2 mm, ML = ±1.8 mm, DV = −7.0 mm) and NAc shell (AP = +1.6 mm, ML = ±0.7 mm, DV = −7.0, −6.6, −6.2 mm), as per the atlas of Paxinos and Watson [17]. Then the toxin (0.4 µl per side) was injected with nitrogen gas pulses of 20–40 psi using an air compression system previously described [18]. Control animals were injected with saline into NAc core or shell. After two additional minutes, the pipette was slowly withdrawn and the animals were then implanted with electrodes for recording electroencephalogram (EEG) and electromyogram (EMG).

**Figure 3:**
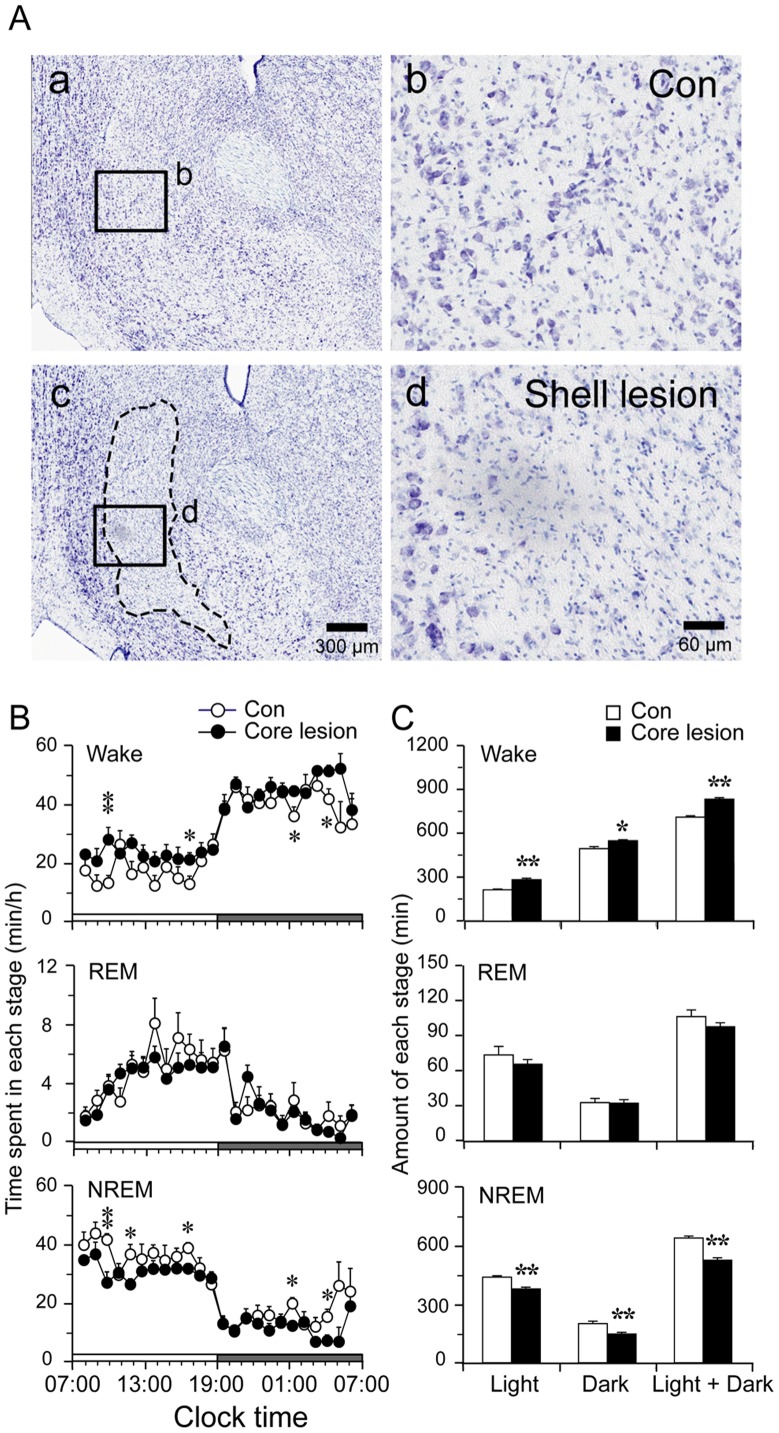
NAc shell lesion increases wakefulness. A: Representative thionin-stained coronal sections show intact control (a, b) and lesion (c, d) (b, d: high-magnification views of the rectangular areas marked in “a” and “c”, respectively). Dotted lines in “c” outline the lesion area that matches the NAc shell. Scale bars are 300 µm in “a” and “c”; 60 µm in “b” and “d”. B: The hourly amount of wakefulness, REM and NREM sleep of control and NAc shell lesioned rats. Each circle represents the hourly mean ± SEM of each stage. C: Total time spent in wakefulness, REM and NREM sleep during the light and dark period and 24-hours. *p<0.05, **p<0.01.

### EEG/EMG Recording and Sleep Scoring

Rats were chronically implanted with EEG and EMG electrodes for polysomnographic recordings. The implant consisted of 2 stainless steel screws (1 mm diameter) inserted through the frontal (AP = +2 mm, ML = +3 mm) and parietal bones (AP = −4 mm, ML = +3 mm), and a stainless steel screw (1.5 mm diameter) inserted on the left frontal bone (AP = +3 mm, ML = −3 mm) as a reference electrode. Two wire electrodes served as EMG electrodes were placed into the neck muscles. All electrodes were attached to a connector and fixed to the skull with dental cement.

**Figure 4:**
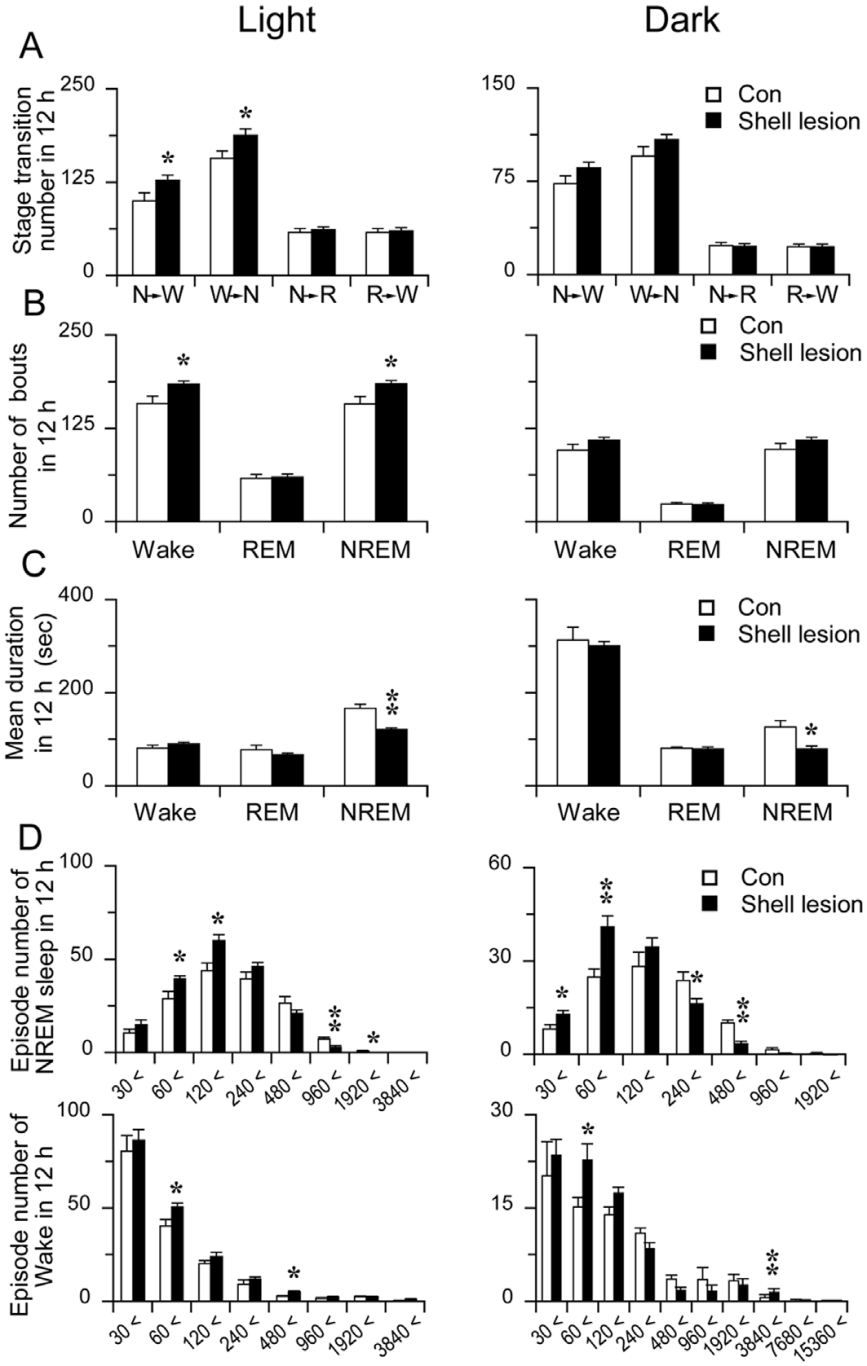
NAc shell lesion causes sleep fragmentation. A: Sleep-wake stage transitions during the light and dark period (N, W and R represent NREM, wakefulness and REM sleep, respectively). B and C: The number of bouts (B) and mean durations (C) during the light and dark periods. D: Number of NREM sleep and Wake bouts at different ranges of episode duration during the light and dark periods.*p<0.05, **p<0.01.

The recording of EEG and EMG were performed by means of a slip ring, designed so that behavioral movement of the animal would not be restricted. After a 7 d recovery period, the animals were housed individually in transparent barrels and habituated to the recording cable for 3 d before polygraphic recordings. EEG/EMG signals were amplified and filtered (EEG: 0.5–30 Hz, EMG: 20–200 Hz), then digitized at a sampling rate of 128 Hz, and recorded using SLEEPSIGN software [19]. When completed, polygraphic recordings were automatically scored off-line by 10 s epochs as wakefulness, REM, and NREM sleep by SLEEPSIGN according to standard criteria [20]. As a final step, defined sleep-wake stages were examined visually, and corrected, if necessary.

**Figure 5:**
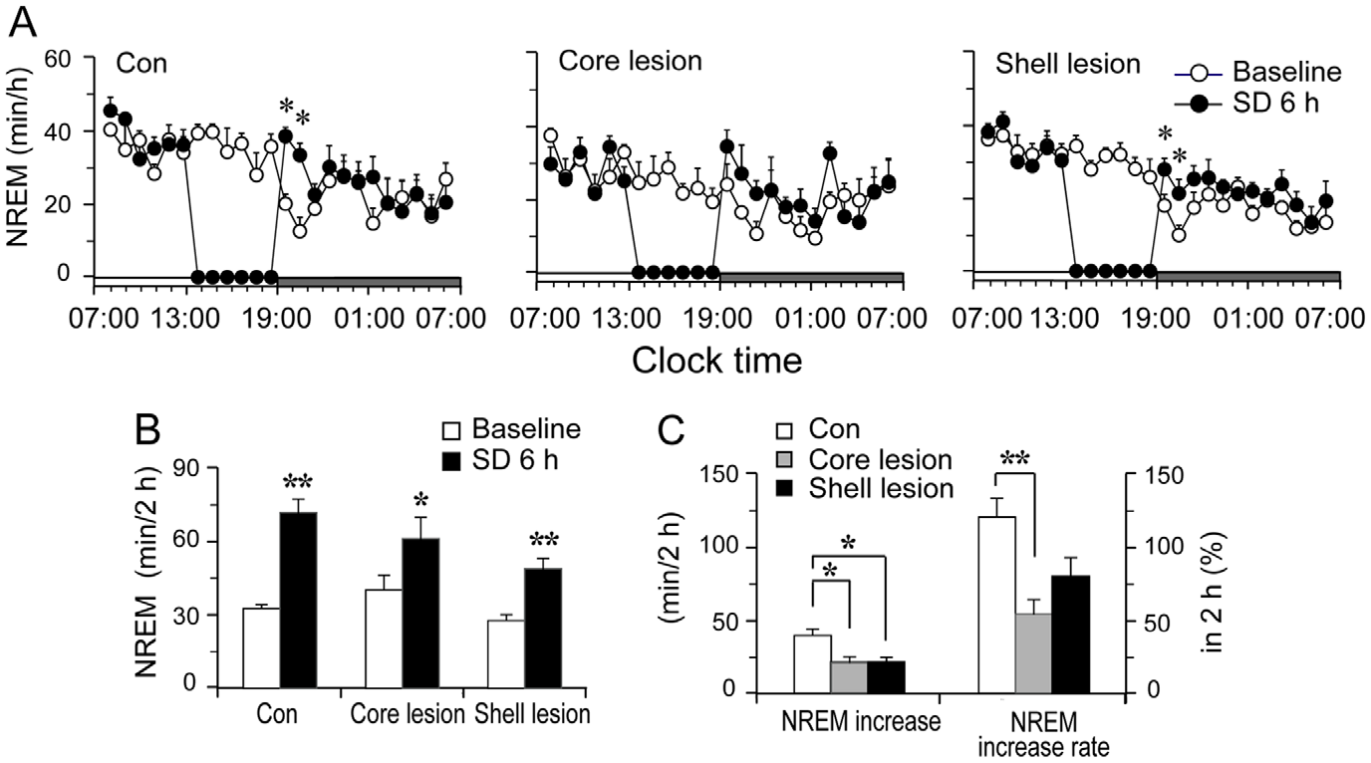
NAc lesions reduce sleep rebound. A: Time-course of NREM sleep in baseline and SD of control, NAc core and shell lesioned rats. SD was between 13∶00 and 19∶00. Each circle represents the hourly mean ± SEM of NREM sleep. B: Total time spent in NREM sleep in 2 h after SD. C: The absolute increased NREM sleep amounts and the percentage of NREM sleep increase in 2 h after SD of each group. *p<0.05, **p<0.01.

### Sleep Deprivation

Rats were adapted in recording chambers for 3 days, and monitored for EEG and EMG for 2 consecutive days. The first day served as the baseline day; and on the second day the animals were subjected to a total sleep deprivation for 6 h (from 13∶00 to 19∶00) by lightly tapping via a soft tissue ball [21].

### Pharmacological Treatments

Modafinil (Sigma-Aldrich) was dissolved in sterile saline containing 10% DMSO and 2% (w/v) cremophor immediately before use and administered intraperitoneally (i.p.) at 9 A.M. on the experimental day at a dose of 90 mg/kg. For baseline date, rats were injected i.p. with vehicle at 9 A.M.

### Histochemistry

Animals were deeply anesthetized with 500 mg/kg of chloral hydrate and transcardially perfused with 50 ml saline, followed by 250 ml of neutral phosphate buffered 10% formalin. The brains were removed, cryoprotected in 20% sucrose at 4°C overnight and then sectioned at 30 µm on a freezing microtome in four series. One series of sections were processed for Nissl staining as described previously [11] to evaluate the extent of the lesions.

### Statistical Analysis

The data were presented as the mean ± standard error of mean (SEM). The statistical significance of time course data for sleep–wake profiles, sleep amount, stage transition, the number of each stage bouts and mean duration were assessed by two-tailed unpaired t-test or one-way ANOVA followed by Dunnett’s post hoc test. In all cases, P<0.05 was taken as the level of significance.

**Figure 6:**
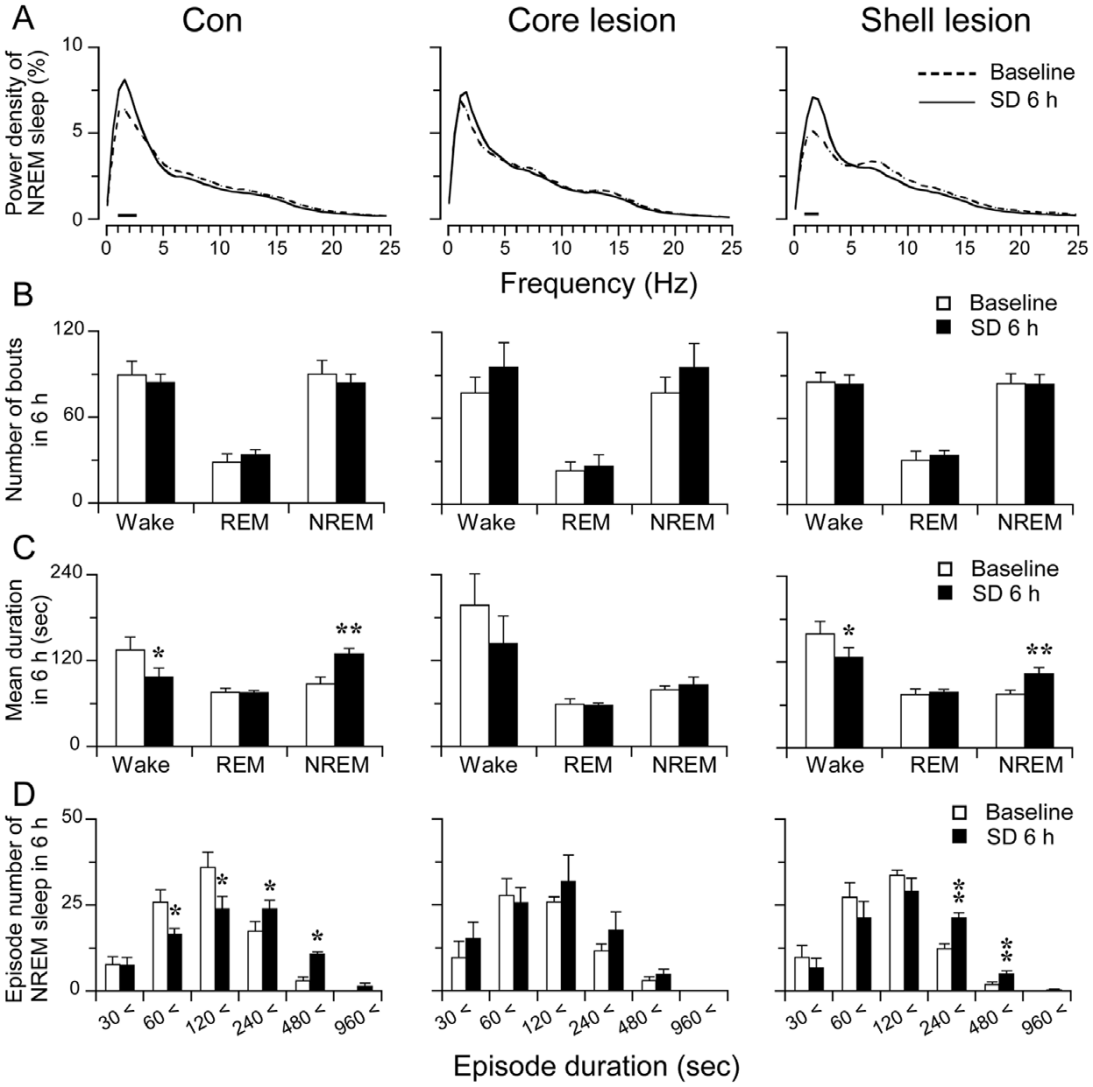
Power spectrum and stage analysis of sleep homeostatic response. A: Relative average EEG power density of NREM sleep during the first 6 hrs after SD. The horizontal bars indicate statistical difference (p<0.05) between SD and baseline of each group. B: Total number of wake, REM and NREM sleep bouts in the first 6 hrs after SD. C:Mean duration of wake, REM and NREM sleep in the first 6 hrs after SD. D: Changes in number of NREM sleep bouts at different ranges of episode duration in the first 6 hrs after SD. *p<0.05, **p<0.01.

## Results

### Lesions of NAc Core and Shell cause a Robust Increase in Wakefulness

Consistent with our previous data [11], we observed that cell-specific lesions confined to NAc core (N = 10,typical examples of coronal sections photographs were shown in Fig. 1A and B) produced a robust 26.5% (838.9±89.7 versus 663.2±113.1 min in the control group, p<0.01) wake increase accompanied by a reduction in REM and NREM sleep per day (Fig. 1C and D), especially during the light period. NAc core lesions also disrupted sleep pattern, resulting more frequent sleep-wake state transition (Fig 2. A), or more wake and NREM sleep bouts and shorter duration of NREM sleep (Fig. 2B and C). The mean duration of NREM sleep was 28.5% (106.1±8.0 versus 148.5±4.5 sec, p<0.01) shorter than the control (Fig. 2C). Although the mean wake duration showed a tendency in lengthening, it did not reach statistical significance (p>0.05). We further calculated the distribution of NREM sleep and wake bouts and found that NAc core lesions particularly had more NREM sleep bouts in the ranges of 30–60 and 60–120 s but less in ranges of 240–480 and 480–960 s during the light period than control (Fig. 2D). The distribution of wake bouts did not show significant changes.

NAc shell lesion group (n = 9, typical photographs of histology of NAc shell lesions were shown in Fig. 3A) showed a 17.4% increase in wakefulness (820.6±9.2 versus 699.0±10.2 in the control group, p<0.01) (Fig. 3B and C), accompanied by a reduction in total NREM sleep. NAc shell lesions caused sleep fragmentation, more NREM sleep bouts with shorter average duration than control (Fig. 4). However, REM sleep change in term of duration and bout number did not reach statistical significance.

### NAc Core and Shell Lesion Reduces Response of Sleep Homeostasis

To determine whether the NAc is involved in sleep homeostatic regulation, we performed a six hour SD from 13∶00 to 19∶00 in NAc core, NAc shell lesion group, and control group. The sleep time, EEG power spectra and the changes of characteristics of sleep-wake episodes during NREM sleep in 6 hrs after SD over the baseline of the same period of prior day were used to determine and quantify the sleep rebound. Fig. 5A and B summarized the time-courses of the hourly amounts of NREM sleep, and the cumulative amounts of NREM sleep for two hours after SD. Control rats, following the SD, showed a marked increase in NREM sleep in first two hours (Fig. 5A, left panel). NAc core lesion group showed significant NREM sleep rebound in the first two hours (p<0.05, Fig. 5B), but this NREM sleep increase was significantly lower than the control (Fig. 5A, middle panel). Similarly, the NAc shell lesion group showed a significant sleep rebound, but the increase was significantly less than the control (p<0.05, Fig. 5C, left panel). The percentage increase of sleep rebound in the first two hours was 119.9±0.12% (p<0.01) in control group, 53.7±0.37% (P<0.05) in NAc core lesion group, and 79.6±0.14% (p<0.01) in NAc shell lesion group (Fig. 5B and C). Of these changes, NAc core lesion group showed the least rebound (Fig. 5C, right panel). REM sleep did not increase during the first hour after SD in control rats.

We further analyzed the EEG power spectra during NREM sleep in 6 hrs after SD among control, core lesion and shell lesion rats, The power of each 0.5 Hz bin was first averaged across the sleep stages individually and then normalized as a group by calculating the percentage of each bin from the total power (0–24.5 Hz) of the individual animal. As shown in Fig. 6A, following SD, EEG power density significantly increased in the frequency range of 1–2.5 Hz in control rats, and in the frequency range of 1–2 Hz in shell lesioned rats, whereas core lesioned rats did not show a significant change.

During the 6 hrs after SD, compared with their own baseline EEG, the bouts of each stage were not changed (Fig. 6B), while in control rats, the mean duration of wake episodes was decreased 25.3% ±9.1% (p<0.05), meanwhile the mean duration of NREM sleep episode was significantly increased by 51.1% ±13.3% (p<0.01). The NAc shell lesion group showed similar but less changes than the control ones. NAc core lesion group did not show changes in mean duration of wake and NREM sleep episodes (Fig. 6C).

To better understand sleep-wake profile following SD, we calculated distribution of NREM sleep bout duration (Fig. 6D). Control group showed less in the number of bout duration range of 30–60 and 60–120 sec but more in the range of 120–240 and 240–480 sec during the 6-hr sleep recovery period than that of the baseline. NAc shell lesioned group showed similar changes as control group, while core lesion group showed reduced effects.

### NAc Core Lesion Blocks Modafinil-induced Arousal

**Figure 7:**
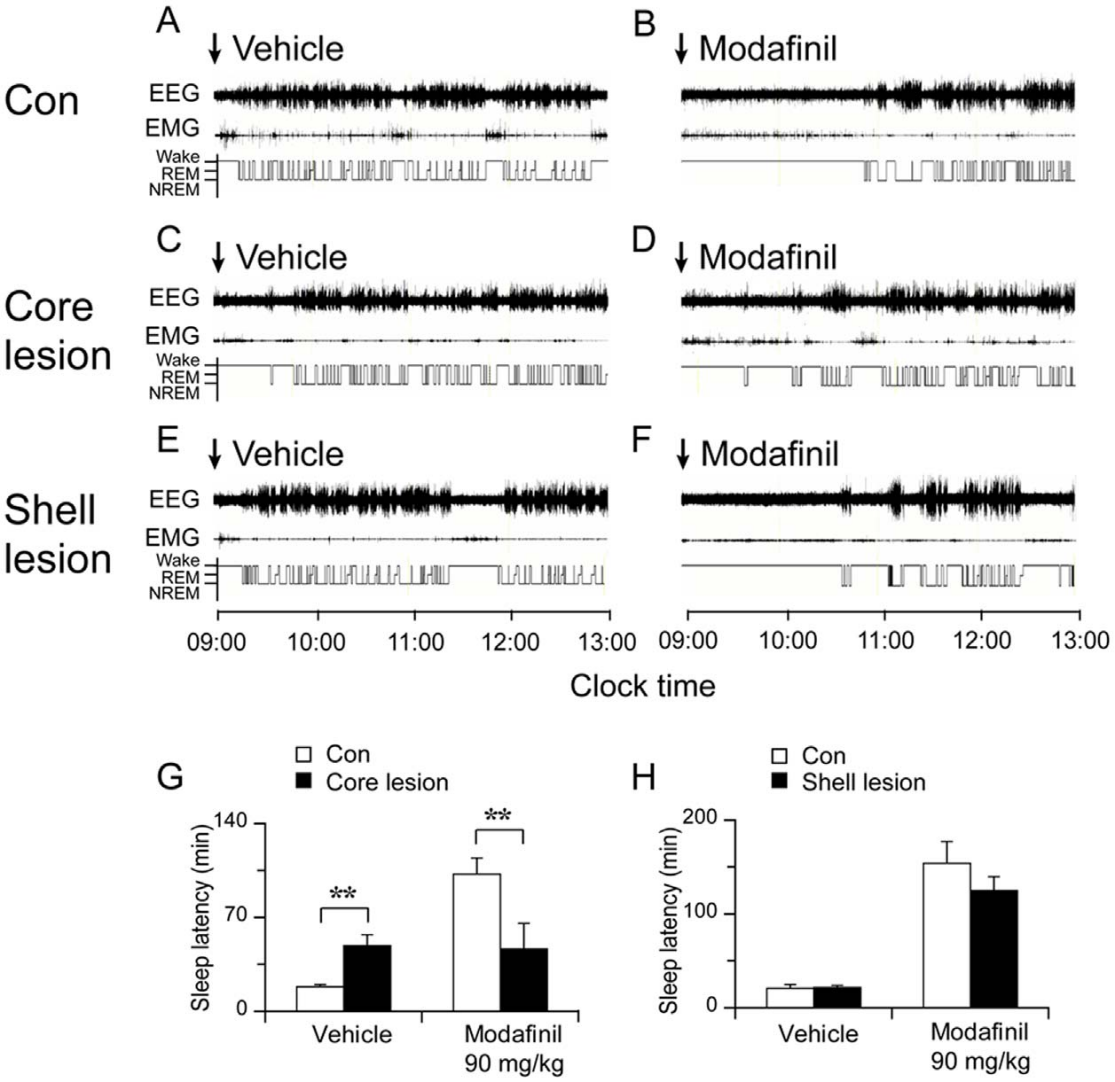
NAc core lesion reduces sleep latency following modafinil administration. Examples of polygraphic recordings and corresponding hypnograms in control, NAc core and shell lesioned rats treated with vehicle (A, C and E) and modafinil at the dose of 90 mg/kg (B, D and F). The arrows indicate the time of vehicle or modafinil injection. G and H: Effect of modafinil on NREM sleep latency in NAc core lesioned, shell lesioned and their control rats. *p<0.05, **p<0.01.

**Figure 8:**
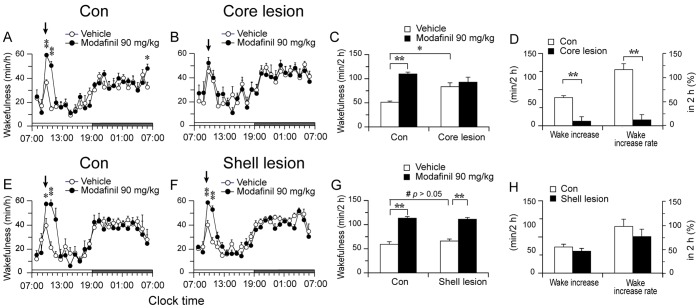
NAc core lesion blocks modafinil-induced arousal. A, B, E and F: Time course changes of wakefulness produced by i.p. administration of vehicle or modafinil (90 mg/kg). Each circle represents the hourly mean ± SEM. Arrows indicate the injection time (9 A.M.). C and G: Total time spent in wakefulness during two hours after the vehicle and modafinil administration. D and H: The increased wakefulness (min) and the percentage of wake increase in two hours after modafinil administration of each group. *p<0.05, **p<0.01.

In order to determine whether the NAc core or shell mediates arousal effects of modafinil, we injected vehicle or modafinil (90 mg/kg) at 9∶00 A.M. in three groups of rats. Fig. 7A–F shows examples of polygraphic recordings and corresponding hypnograms for a rat of each group treated with vehicle or modafinil. To our surprise, vehicle injection in NAc core lesioned animals significantly induced more wakefulness than the control rats (83.7±8.2 versus 51.4±2.5 min in the control group, p<0.05, Fig. 8C). Modafinil induced continuous wakefulness for about 2 hrs in control and NAc shell lesioned rats (Fig. 7B and F; Fig. 8A, E and F), which was significantly longer than vehicle injection. In the NAc core lesioned rats, modafinil produced about 1.5 hrs continuous wakefulness, and which was not significantly different from its vehicle injection (Fig. 7C and D; Fig. 8B and C). Next, we investigated the sleep latency in rats injected with modafinil. Sleep latency was defined as the time from the injection of modafinil or vehicle to the appearance of the first NREM sleep episode lasting for at least 20 s [22]. As shown in Fig. 7G and H, modafinil significantly prolonged the NREM sleep latency in control group. In core lesioned group, sleep latency of modafinil was significantly decreased. Interestingly, vehicle injection also produced similar sleep latency as the modafinil injection (Fig. 7G) in core lesioned group. Shell lesion did not affect the effect of modafinil on sleep latency (Fig. 7H).

The net wake increase in two hours after modafinil injection vs vehicle injection were 59±4.0 min in control group (Fig. 8C and D). The percentage increase of wakefulness after modafinil over vehicle injection was 116.8±12.1% in control group and 12.3±10.9% in NAc core lesioned group (Fig. 8C and D).

Rats with NAc shell lesions showed similar arousal response and NREM sleep latency to modafinil as the control rats (Fig. 8E–H).

## Discussion

In the present study, we demonstrated that lesions of both NAc core and shell produced a significant wake increase, and reduced sleep homeostatic response, with NAc core lesions showing a strong effect. NAc core lesions but not NAc shell lesions blocked arousal response to modafinil.

Our previous observation [11] showed that bilateral striatal lesions resulted in a significant reduction in time spent in wakefulness, as well as fragmentation of both sleep and wakefulness. However, when the striatal lesions include the NAc, their effect on wakefulness is attenuated. Consistent with this observation, lesions restricted to the whole NAc produce an increase in wakefulness and a reduced duration of bouts of NREM sleep. These findings suggest that the dorsal and ventral striatum play opposing roles in sleep–wake regulation: the caudate–putamen (or dorsal striatum) enhances wakefulness whereas the NAc (or ventral striatum) promotes sleep. The present study aimed to elucidate the role of core or shell in sleep-wake regulation. We used a low concentration of ibotenic acid (1.0%, 400 nl per side) to make the core lesion more restricted, whereas the former study used a high concentration (10%, 45 nl per side) of ibotenic acid which made core lesion to partially damage the shell. In general, NAc core and shell lesion rats exhibited a similar phenotype in increased wakefulness, and sleep fragmentation (more sleep-wake transitions, reduced NREM sleep mean duration, and increased episode numbers for wake and sleep). These changes mainly occurred during the light period, indicated that NAc core and shell lesions resulted in the instability of NREM sleep under baseline conditions, especially in their inactive period.

After a 6-hr SD, the control rats showed a significant sleep rebound as indicated by an increase of NREM sleep amount with enhanced delta EEG power and increased mean duration and the number of long bouts during the first 6 hr period post SD, However NAc core lesioned rats did not show the prolongation of NREM sleep and enhancement of sleep intensity and consolidation. NAc shell lesion group showed similar but less prominant changes, compared with the control ones. The reduced sleep rebound after SD in NAc core and shell lesion suggests that NAc is involved in sleep homeostatic regulation.

Unlike psychostimulants such as methamphetamine, modafinil does not have strong psychological dependence and abused tendency [23]–[26]. On the other hand, like pyschostimulants, modafinil’s arousal property depends on the dopamine system [27], [28]. Mice with DAT knockout that have high extraceullar dopamine do not produce a wake response following modafinil administration [15], and dopamine D_2_ receptor knockout mice treated with a D_1_ receptor antagonist abolish the arousal effects by modafinil [16]. Although orexin and histamine systems are activated by modafinil [29], [30], they may not be essential for the arousal effects of modafinil as orexin and histidine decarboxylase (an enzyme for histamine synthesis) knockout mice have a normal arousal response to modafinil [15], [31], [32]. Core lesion but not shell lesion abolished the arousal effects of madafinil, suggesting that dopamine receptors expressed in the core are essential for the arousal effects of modafinil.

The NAc core mediates arousal effects of modafinil. Interestingly, adenosine A_2A_ receptors in the NAc shell but not in NAc core play a pivotal role in regulation of caffeine-induced arousal [12]. It has been established that caffeine induces arousal via adenosine system, but not dopamine system [12], [15], [33], [34]. Thus the NAc may be the hub that mediates multiple neurotransmitters including adenosine and dopamine for sleep-wake control [12], [35], [36].

How the NAc regulates sleep is not completely clear. The NAc has GABAergic projections to a wide range of targets, including the ventral pallidum, the lateral hypothalamus, the parabrachial nucleus and the VTA, that may contribute to wakefulness [37]. Therefore, it can be hypothesized that NAc activation exerts inhibitory effects on important arousal systems and promotes sleep. Although both core and shell are important in sleep-wake regulation, the mechanisms on different roles of these two parts in homeostasis regulation and in the arousal effects of modafinil/caffeine remain to be elucidated.
